# Association Between Spiritual Care Competence and Palliative Care Quality Among Palliative Care Nurses: A Cross-Sectional Study

**DOI:** 10.3390/healthcare14172796

**Published:** 2026-09-01

**Authors:** Neşe Ataman Bor, Kamber Sümer, Nesrullah Ayşin

**Affiliations:** 1 Department of Nursing, Faculty of Health Sciences, Hakkari University, Hakkari 30000, Turkey; 2 Health Services Vocational School, Hakkari University, Hakkari 30000, Turkey; nesrullahaysin@hakkari.edu.tr

**Keywords:** palliative care, spiritual care, nursing, spiritual care competence, quality of care

## Abstract

Background/Objectives: Spiritual care is an important component of holistic palliative care. This study aimed to examine the association between spiritual care competence and perceived quality of palliative nursing care among nurses providing palliative care across different clinical settings. Methods: A descriptive, cross-sectional, correlational study was conducted among 160 nurses providing palliative care in palliative care units, oncology wards, and intensive care units between 1 September and 31 October 2024. Data were collected using a Personal Information Form, the Palliative Care Quality Scale for Nurses (PNCQS), and the Spiritual Care Competence Scale (SCCS). Descriptive statistics, independent-samples t-tests, one-way analysis of variance, Pearson correlation analysis, and multiple linear regression analyses were performed. Statistical significance was set at *p* < 0.05. Results: The mean age of the nurses was 30.43 ± 8.95 years; 77.5% were female, 49.4% had received palliative care training, and 21.9% had received spiritual care training. Overall, 21.9% considered themselves competent in providing spiritual care, while 23.8% identified excessive patient workload as a barrier to providing spiritual care. The mean PNCQS and SCCS scores were 75.08 ± 10.15 and 92.8 ± 12.1, respectively. PNCQS scores were significantly higher among female nurses and nurses with postgraduate education. PNCQS and SCCS scores were weakly but significantly positively correlated (r = 0.161, *p* = 0.042). In the multiple linear regression analysis, postgraduate education, gender, and SCCS scores were significantly associated with PNCQS scores. The model explained 15.0% of the variance in PNCQS scores (adjusted R^2^ = 0.134). Conclusions: Spiritual care competence was weakly associated with nurses’ perceived quality of palliative nursing care. Given the cross-sectional design and the use of self-reported measures, this association should not be interpreted causally. The limited explanatory power of the regression model suggests that perceived quality of palliative nursing care is influenced by multiple individual, clinical, educational, and contextual factors. Future longitudinal and multimethod research should examine these factors using objective and patient-centered indicators across diverse clinical settings.

## 1. Introduction

Advances in healthcare and technology have increased life expectancy and contributed to a growing burden of chronic and life-limiting diseases, thereby increasing the global need for palliative care [1]. It is estimated that 56.8 million people require palliative care annually, and this need is expected to increase further as populations age and the burden of chronic diseases continues to rise [2]. Palliative care is therefore recognized as an essential component of health systems and aims to improve quality of life by preventing and relieving suffering through the early identification and management of physical, psychosocial, and spiritual problems [2,3,4,5,6]. Importantly, palliative care is not restricted to older adults or a single clinical setting; it may be required across the life course and in diverse settings, including oncology, adult intensive care, pediatric intensive care, and other services caring for patients with serious or life-limiting conditions [3,6].

As a core component of holistic care, palliative nursing requires nurses to integrate professional knowledge, clinical skills, communication, and appropriate attitudes to address patients’ physical, psychological, social, cultural, and spiritual needs [7,8]. Among these dimensions, spiritual care is recognized as an important component of person-centered palliative care because patients with life-limiting illnesses may experience existential concerns, loss of meaning, spiritual distress, and questions related to illness, death, and hope [9,10,11,12,13]. Spiritual care competence refers to the knowledge, skills, attitudes, and behaviors required by healthcare professionals to recognize and appropriately respond to patients’ spiritual needs. Recent evidence indicates that, although spiritual care is considered an important component of comprehensive nursing care, nurses’ perceptions and competencies regarding spirituality and spiritual care vary and may require further development, with education and training identified as important areas for improvement [14,15].

In Türkiye, spirituality may be closely associated with Islamic beliefs, cultural traditions, and family-centered values, which can influence how individuals experience illness, suffering, death, and bereavement [16,17]. Spiritual needs reported by palliative care patients include hope, inner peace, compassion, prayer, and companionship, highlighting the importance of culturally sensitive spiritual care [18]. Nurses’ approaches to spiritual care may be influenced by both professional education and sociocultural context, requiring sensitivity to patients’ beliefs, values, and individual philosophies of life [16,17]. Consistent with this context, studies conducted in Türkiye have reported challenges in providing spiritual care and have identified education, knowledge, time limitations, workload, and organizational support as important factors influencing spiritual care practices [13,16,17,19,20,21,22]. Recent research among critical care nurses in Türkiye has likewise demonstrated the relevance of spiritual care competence and spiritual care practices in intensive care settings [15].

Perceived quality of palliative nursing care is a multidimensional concept encompassing symptom management, support for patients and families, therapeutic relationships, spiritual support, and continuity of care, as evaluated from the nurse’s perspective [5,8]. The Palliative Nursing Care Quality Scale (PNCQS) was specifically developed to assess good palliative nursing care from the perspective of nursing professionals [5]. The Turkish version of the PNCQS was subsequently validated and demonstrated adequate validity and reliability for assessing nurses’ evaluations of the quality of palliative nursing care in Türkiye [23]. Importantly, the PNCQS does not directly measure patients’ experiences, clinical outcomes, adherence to predefined quality standards, or objectively observed nursing performance. Rather, its scores represent nurses’ self-reported perceptions of the quality of palliative nursing care they provide [5,23]. Accordingly, in the present study, the term “nurses’ perceived quality of palliative nursing care” is used to distinguish this construct from the broader concept of overall palliative care quality.

Although spiritual care competence and quality of palliative nursing care have each been investigated separately, the relationship between nurses’ spiritual care competence and their perceived quality of palliative nursing care remains insufficiently explored. Recent research in Türkiye has examined factors associated with nurses’ perceived quality of palliative care, including difficulties encountered while providing palliative care [24], whereas other studies have investigated spiritual care practices, spiritual health, or spiritual care competence in specific nursing populations [13,15,25]. For example, Öner et al. [24] examined the association between difficulties experienced by palliative care nurses and their perceived quality of palliative care using the PNCQS, while Erbay Dallı [15] investigated the relationship between spiritual care practices and spiritual care competence among critical care nurses in Türkiye using the Spiritual Care Competence Scale (SCCS). Similarly, Kılınç İşleyen and Akbaş [25] examined the relationship between spiritual health and spiritual care competence among nurses. However, these studies did not directly examine whether nurses’ spiritual care competence is associated with their perceived quality of palliative nursing care. Thus, evidence directly addressing the relationship between these two constructs remains limited.

Although spiritual care is an integral component of holistic palliative nursing, spiritual care competence and perceived quality of palliative nursing care represent distinct constructs. The SCCS assesses nurses’ competence in recognizing and responding to patients’ spiritual needs, whereas the PNCQS assesses nurses’ perceptions of the overall quality of palliative nursing care they provide [5,15,23]. Examining these constructs together may provide insight into whether competence in a specific domain of palliative nursing is associated with nurses’ broader perceptions of care quality. This relationship is conceptually relevant because spiritual support constitutes an important component of holistic palliative nursing care, whereas spiritual care competence reflects nurses’ preparedness and capability to provide such care [9,10,11,12,13,14,15]. Nevertheless, the relationship between spiritual care competence and nurses’ perceived quality of palliative nursing care has not been sufficiently examined, particularly in Türkiye and across diverse clinical settings.

Furthermore, palliative care should not be conceptualized as care exclusively provided to older adults or within specialized palliative care units. Patients with serious or life-limiting conditions may require palliative approaches in oncology, adult intensive care, pediatric intensive care, and other clinical settings [2,3,6]. Nurses working in these settings may encounter patients and families experiencing symptom burden, uncertainty, existential concerns, spiritual distress, and end-of-life issues, although the clinical context and patient populations may differ. The need for palliative approaches across different patient populations and clinical contexts has also been emphasized in the broader palliative care literature [1,2,3,4,5,6]. Therefore, examining spiritual care competence and perceived quality of palliative nursing care across diverse clinical settings may provide a broader understanding of how these constructs are related beyond specialized palliative care units.

Given the importance of spiritual care within holistic palliative nursing and the limited evidence regarding its relationship with nurses’ perceived quality of palliative nursing care, the present study aimed to examine the association between spiritual care competence and nurses’ perceived quality of palliative nursing care in Türkiye. By including nurses providing care in different clinical settings, including oncology, adult intensive care, pediatric intensive care, and palliative care units, this study examined the relationship across diverse clinical contexts rather than restricting the investigation to nurses working exclusively in specialized palliative care units. This approach is consistent with the broader understanding of palliative care as an approach applicable across diseases, patient populations, and care settings [1,2,3,4,5,6]. The findings may contribute to a better understanding of the statistical association between spiritual care competence and nurses’ perceived quality of palliative nursing care and may inform future educational and organizational initiatives.

## 2. Material and Method

### 2.1. Study Design

This was a descriptive, cross-sectional, correlational study.

### 2.2. Study Population and Sample

The study population consisted of 254 nurses working in palliative care units, oncology wards, and intensive care units, including surgical, medical, general, coronary, neurology, and pediatric intensive care units, who were registered members of the Palliative Care Nurses Association in Türkiye. To be eligible for membership in the Association, nurses were required to have at least one year of professional nursing experience in clinical settings providing palliative care. Accordingly, eligibility criteria for the present study were membership in the Association and voluntary participation. The Association was contacted to facilitate access to eligible participants. However, for ethical and confidentiality reasons, the researchers were not provided with information identifying the hospitals where Association members worked. The survey link was electronically distributed by the Association manager through the Association’s communication channel.

A non-probability convenience sampling method was used because eligible nurses were accessible through the Association. Given the known population size (N = 254), the minimum required sample size was calculated as 153 nurses using the formula proposed by Morgan (1970) [26], assuming a 95% confidence level and a 5% margin of error.

Data were collected between 1 September and 31 October 2024. Eligible participants were actively employed nurses who routinely provided palliative care as part of their clinical practice during the data collection period. Participants had a mean of 5.96 ± 3.83 years of palliative care experience (median = 6.0; range = 1–12 years). Of the 254 eligible nurses approached, 160 voluntarily participated and completed the questionnaires, yielding a participation rate of 63.0%. The remaining 94 nurses did not participate: 35 declined participation, 18 were on unpaid leave because of childcare responsibilities, 3 were on annual leave, and 38 could not be reached during the data collection period.

### 2.3. Data Collection and Instruments

In the study, the “Personal Information Form”, the “Palliative Care Quality Scale for Nurses (PNCQS)” and “Spiritual care competence scale (SCCS)” were used to collect research data.

#### 2.3.1. Participant Information Form

The Information Form included 22 questions addressing nurses’ sociodemographic characteristics such as age, years of experience, educational status, and whether they had received training in palliative and spiritual care. The items were developed based on previous studies [27,28,29].

#### 2.3.2. Palliative Care Quality Scale for Nurses (PNCQS)

The scale was developed by Zulueta Egea et al. (2020) [29] and its Turkish validity and reliability study was conducted by Mollaoğlu and Boy (2023) [23]. The scale does not have subdimensions and is evaluated based on the total score, with a minimum score of 18 and a maximum score of 90. Higher scores on the scale indicate that nurses perceive the quality of the palliative nursing care they provide as higher. In the study by Mollaoğlu and Boy (2023) [23], the Cronbach’s alpha coefficient of the scale was calculated as 0.92. In this study, the Cronbach’s alpha coefficient of the scale was found to be 0.97.

#### 2.3.3. Spiritual Care Competence Scale (SCCS)

Developed by Van Leeuwen et al. (2009) [30], the validity and reliability of the Turkish version were established by Dağhan et al. (2018) [31]. The assessment tool measures nurses’ spiritual care competence and consists of three sub-dimensions: assessment and implementation of spiritual care, professionalism and patient counseling, and attitude and communication toward the patient’s spirituality. The scale has a total of 27 items and uses a five-point Likert response format (1 = Strongly disagree, 5 = Strongly agree), with a score range of 27–135. In the Turkish validity and reliability study of the scale, the Cronbach’s alpha reliability coefficient was determined to be 0.97 [31]. In the present study, the Cronbach’s alpha coefficient was also found to be 0.97. The Cronbach’s alpha coefficients for the three subdimensions were also high: 0.97 for Assessment and Implementation of Spiritual Care, 0.96 for Professionalism and Patient Counseling, and 0.96 for Attitude and Communication toward the Patient’s Spirituality. These findings indicate excellent internal consistency for both the overall scale and its subdimensions in the present sample.

### 2.4. Data Collection

Data were collected anonymously via Google Forms using an online survey link between 1 September and 31 October 2024. The researchers did not have direct access to the participants’ personal contact information. The survey link was distributed by the manager of the Palliative Care Nurses Association via WhatsApp to all 254 eligible nurses working in palliative care, oncology, and intensive care units.

The Association representative communicated with eligible members regarding participation and, while maintaining confidentiality and without disclosing any identifying information to the researchers, provided the research team with aggregated information regarding participation status and the reasons reported by members who did not participate. Accordingly, the reasons for non-participation among the 94 eligible nurses were obtained through the Association representative rather than through direct contact by the researchers. “Could not be reached” referred to eligible members for whom no response or participation confirmation was received by the Association representative during the data-collection period and did not indicate any attempt by the researchers to contact these individuals.

The survey did not require Google account sign-in, and email address collection was disabled; no direct or indirect identifiers, including names, email addresses, phone numbers, or IP addresses, were collected. The questionnaire was designed using a page-transition structure. The first page provided detailed information about the study purpose, procedures, voluntary nature of participation, confidentiality, and participants’ right to withdraw before submission without consequences. Electronic informed consent was obtained through a mandatory “voluntary participation approval” checkbox, and only participants who provided consent could proceed to the questionnaire. On the subsequent page, participants were asked whether they had previously completed the survey. Those who selected “No” were directed to the sociodemographic questions and scale items. This procedure was implemented to reduce the possibility of duplicate responses while maintaining participant anonymity.

Convenience sampling was used to recruit participants because eligible nurses were accessible through the Association. Completion of the questionnaire required approximately 10–15 min.

### 2.5. Data Analysis

Data were analyzed using IBM SPSS Statistics for Windows, Version 25.0 (IBM Corp., Armonk, NY, USA) [32]. During the analysis process, descriptive statistics, reliability assessments, and tests for normality assumptions were conducted. Normality was evaluated by examining whether skewness and kurtosis values fell within the ±2 range [33,34]. Skewness and kurtosis values within this range indicate that the data meet the assumption of normal distribution, supporting the use of parametric tests (PNCQS: Skew. = −0.623; Kurt. = −0.894) (SCCS: Skew. = −0.234; Kurt. = −0.583). For data analysis, multiple linear regression analyses, ANOVA, Pearson correlation, and independent samples *t*-tests were used. A significance level of *p* < 0.05 was accepted for all statistical tests.

To evaluate potential confounding, sensitivity analyses were conducted by comparing the final model against the fully saturated model (Supplementary Material S1, Table S1). The inclusion of all theoretical covariates (e.g., unit, experience, barriers) did not meaningfully alter the primary SCCS estimate (B_(full)_ = 0.135, *p* = 0.037 vs. B_(final)_ = 0.127, *p* = 0.044), supporting the robustness of the observed association to adjustment for the measured covariates.

## 3. Results

Table 1 presents sociodemographic and clinical characteristics. The mean age was 30.43 ± 8.95 years; 67.1% were under 35 years old, 77.5% were female, 64.6% held a bachelor’s degree, and 49.4% had received palliative care training. Among the participants, 36.3% had 11 years or more of nursing experience, 21.9% had received training on spiritual care, and 21.9% considered themselves competent in providing spiritual care. Additionally, 23.8% reported that one of the barriers to providing spiritual care was having too many patients.

The comparison of the PNCQS scores based on the personal and working conditions of nurses working in palliative care units is presented in Table 2. Nurses with postgraduate education had significantly higher mean PNCQS scores than those with bachelor’s degree and those with associate degree or less, with a moderate effect size (Welch’s F = 25.0, *p* < 0.001, η^2^ = 0.10). The total PNCQS score was statistically significant for gender with a small-to-moderate effect size; female nurses scored higher than male nurses (t = 2.778; *p* = 0.006, η^2^ = 0.046). Although nurses who had received palliative care training, had 11 years or more of professional experience, and were aged 36 or older had relatively higher mean total scores on the PNCQS, the differences between the groups were not statistically significant. As shown in Table 2, the mean PNCQS score of female nurses was moderately higher than that of male nurses (t = 2.78, *p* = 0.006, d = 0.53). No statistically significant differences were observed between the other variables and the scale scores (*p* > 0.05).

Table 3 presents the mean scores of the PNCQS (75.08 ± 10.15) and the SCCS (92.8 ± 12.1), along with their subdimensions: assessment and implementation of spiritual care (21.03 ± 3.40), professionalism and patient counseling (51.35 ± 8.11), and attitude and communication toward patients’ spirituality (21.76 ± 4.18). A weak positive correlation was observed between spiritual care competence and perceived quality of palliative nursing care (r = 0.161, *p* = 0.042).

A multiple linear regression analysis was performed to determine which variables statistically predicted participants’ PNCQS scores (Table 4). To adhere to the principle of parsimony, the final model was constructed using a two-step selection process based on preliminary univariate results (Table 2 and Table 3). To validate this approach, a fully saturated model including all theoretically relevant measured covariates was also executed (detailed in Supplementary Material S1). In the fully saturated model, gender, educational level, and SCCS remained statistically significant predictors of PNCQS. Comparison of the saturated and final parsimonious models showed that adjustment for the measured covariates resulted in only a small change in the SCCS coefficient (B = 0.135 vs. B = 0.127), suggesting that the primary association was relatively stable across model specifications. Dummy coding was applied for categorical variables, with female set as the reference category for gender. For education level, postgraduate education was coded as a dummy variable, with participants with an associate degree or less and a bachelor’s degree combined as the reference category (Table 4).

The regression model was statistically significant (F (3.156) = 9.19, *p* < 0.001) and explained 15% of the variance in PNCQS scores (adjusted R^2^ = 0.134). No multicollinearity issues were detected (VIF < 5, Tolerance > 0.10), and the Durbin–Watson value was within the acceptable range (1.5–2.5).

Education level was the strongest positive predictor of PNCQS scores (B = 8.813, 95% CI [4.61, 13.01], β = 0.311, t = 4.145, *p* < 0.001), with participants holding postgraduate degrees scoring significantly higher than the reference group. Gender had a significant negative effect on PNCQS scores (B = −5.182, 95% CI [−8.757, −1.606], β = −0.214, t = −2.862, *p* = 0.005), indicating that male participants scored on average 5.18 points lower than females (reference category). SCCS scores were also a significant positive predictor (B = 0.127, 95% CI [0.003, 0.252], β = 0.153, t = 2.027, *p* = 0.044), with each one-point increase in SCCS associated with a 0.127-point increase in PNCQS scores.

Regression diagnostics for the final parsimonious model confirmed that all multivariate assumptions were robustly satisfied. The standardized residuals demonstrated a mean of 0.000 and a standard deviation of 0.991. Furthermore, the standardized residual values ranged between −2.466 and 1.848; since no observations exceeded the critical threshold of (±3.0), the final model was verified to be free from any distorting influential outliers. (The figures are presented in Supplementary Material S2.)

## 4. Discussion

This study examined the statistical association between nurses’ spiritual care competence and their perceived quality of palliative nursing care. Nurses reported relatively high levels of perceived palliative nursing care quality, consistent with findings by Zulueta Egea et al. [29] and Öner et al. [5,24,26], whereas Dong et al. and Zhang et al. reported moderate and low levels, respectively [35,36]. These differences may reflect variations in clinical settings, nurse characteristics, organizational conditions, measurement instruments, and cultural contexts. Because the Palliative Nursing Care Quality Scale (PNCQS) assesses nurses’ self-reported perceptions rather than objective clinical outcomes or patient-reported experiences, the present findings should be interpreted specifically as perceived quality of palliative nursing care.

In this study, nurses with postgraduate education had significantly higher mean PNCQS scores. Similarly, Öner et al. (2025) [24] reported significantly higher PNCQS scores among nurses with postgraduate education [26]. However, given the cross-sectional design, these findings do not establish that postgraduate education improves perceived palliative nursing care quality. The observed difference may partly reflect factors associated with educational level, such as professional experience, palliative care experience, or training [26]. In addition, Košanski et al. (2026) reported that nurses working in specialized palliative care continued to have professional development needs despite their formal educational preparation, highlighting that formal education alone may not fully capture nurses’ ongoing competency development needs [37]. Recent evidence from Lähteenmäki et al. (2025) showed improvements in self-assessed palliative care competence following a structured specialist education programme [38]. Nevertheless, their pretest–posttest design and specific educational intervention differ from the present cross-sectional comparison. Thus, postgraduate education should be interpreted as a characteristic associated with higher PNCQS scores rather than as a causal determinant of perceived palliative nursing care quality. Future longitudinal or quasi-experimental studies should examine whether this association persists after accounting for professional experience, palliative care experience, training, and care setting.

Female nurses also reported significantly higher PNCQS scores, consistent with previous findings and the review by Rabiei Vaziri et al. [39]. However, observed gender differences may be shaped by educational and clinical experiences, professional roles, communication patterns, and contextual expectations rather than reflecting an intrinsic effect of gender [40,41]. Gender should therefore be regarded as a contextual characteristic associated with perceived palliative nursing care quality rather than a causal determinant.

The mean SCCS score was 92.8 ± 12.1, indicating relatively high self-reported spiritual care competence. This finding is broadly consistent with Kalkım et al. (2019) [28], whereas Çelik and Sivrikaya (2024) [26] reported moderate levels, and other studies reported lower levels [39]. Differences may be related to educational background, professional experience, clinical setting, cultural context, and opportunities for spiritual care education and practice. A 2025 systematic review and meta-analysis identified educational background, spiritual care education, working hours, work environment, hospital characteristics, nursing experience, professional position, emotional intelligence, and empathy as factors associated with spiritual care competence [42]. However, because the evidence was predominantly cross-sectional, these factors should be considered correlates rather than causal determinants. Recent Turkish evidence also supports the potential value of structured educational approaches [43].

The findings showed a weak but statistically significant positive association between spiritual care competence and perceived palliative nursing care quality (r = 0.161). This finding should be interpreted cautiously in light of the constructs assessed. The SCCS measures a relatively specific competence in recognizing and responding to spiritual needs, whereas the PNCQS assesses a broader, multidimensional construct encompassing symptom management, family and caregiver support, therapeutic relationships, spiritual support, and continuity of care [29]. Thus, the observed association may partly reflect the fact that spiritual care competence represents one specific professional capacity within the broader construct of palliative nursing care quality. However, the present cross-sectional data do not allow us to determine whether the modest magnitude of the association is attributable to this conceptual distinction or to other individual, clinical, organizational, or measurement-related factors.

Because both constructs were assessed simultaneously using self-reported measures, shared method variance and response-related influences cannot be excluded. Moreover, self-reported competence may not fully reflect the extent to which spiritual care is translated into everyday nursing practice. Accordingly, the weak association observed in this study should not be interpreted as evidence that spiritual care competence has a limited role in palliative nursing care quality. Rather, conceptual non-equivalence between the two measures represents one possible explanation that should be examined in future research. Documentation-based approaches may provide a complementary perspective to that provided by self-reported measures. In particular, natural language processing and semantic mapping of free-text nursing documentation may help identify and standardize spiritual and relational nursing activities that are not adequately captured by structured questionnaires [44]. Vanalli et al. (2023) demonstrated the feasibility of using these approaches to identify and standardize unstructured nursing activities documented in electronic health records, highlighting their potential for capturing aspects of nursing practice that may otherwise remain under-recognized [44].

This interpretation is also consistent with recent European and particularly Italian evidence emphasizing that nursing care complexity is multidimensional and cannot be adequately understood through isolated nursing activities [45,46,47,48]. Moran et al. (2024) highlighted the interconnected clinical, relational, supportive, and coordinating roles of nurses in palliative care [45]. Stalpers et al. (2025) conceptualized nursing work as an “entangled” set of interconnected activities [46], while Fabrizi et al. (2024) showed that medical classification and staffing allocation systems may not fully capture actual nursing care complexity and demand [47]. Reguera-Carrasco et al. (2025) further identified professional experience, communication, professional characteristics, and complex clinical activities as important components of nurse-related care complexity [48].

Recent Italian evidence further strengthens this perspective. In a prospective one-year multicentre cohort study conducted in two Italian hospitals, Cocchieri et al. (2025) found that higher nursing complexity was associated with increased mortality [49]. More recently, Cocchieri et al. (2026) demonstrated substantial variability in nursing diagnoses and nursing actions both across and within diagnosis-related groups among 14.169 adult hospitalizations, indicating that medically similar patients may nevertheless have markedly different nursing care requirements [50]. Together, these findings support the view that nursing complexity is clinically meaningful and multidimensional.

Within this framework, perceived palliative nursing care quality may be understood as emerging from multiple interacting clinical, relational, organizational, and patient-related factors. Spiritual care competence may therefore contribute to, but does not fully determine, the broader quality of palliative nursing care. The present finding should consequently be interpreted as evidence of a weak positive association, while the conceptual distinction between a specific professional competence and a broader multidimensional quality-of-care construct should be regarded as a plausible explanatory perspective rather than as an explanation established by the present data.

The finding that workload was the most frequently reported barrier to spiritual care also warrants attention. However, workload was assessed as a self-reported categorical barrier rather than with a validated continuous measure, and objective indicators such as staffing adequacy, nurse-to-patient ratios, patient acuity, organizational support, teamwork, and missed nursing care were not included in the regression model. Therefore, the present study cannot determine the independent contribution of these factors to perceived palliative nursing care quality or establish whether they confound, moderate, or mediate the observed association. The finding should consequently be interpreted as nurses’ perception of workload as a barrier to spiritual care rather than as evidence that workload or organizational conditions are determinants of perceived palliative nursing care quality. Nevertheless, evidence identifying working hours, work environment, hospital characteristics, professional position, and nursing experience as correlates of spiritual care competence [46], together with research on nursing complexity [46,47,48], supports examining these contextual factors in future studies.

Another noteworthy finding was that nurses who had received palliative care training did not report significantly higher PNCQS or SCCS scores. This result may partly reflect the binary assessment of training, which did not capture its duration, content, recency, intensity, educational method, or clinical applicability. Thus, “having received training” may represent substantially different educational experiences. Lähteenmäki et al. (2025) reported improvements in nurses’ self-assessed palliative care competence following a structured specialist programme [38], while Karabey et al. (2025) examined an innovative educational and compassionate-care approach to spiritual care competence [43].

Importantly, spiritual care training itself was also not significantly associated with SCCS scores in the present study. This finding should be interpreted cautiously because spiritual care training was assessed only as a binary variable and therefore did not distinguish between the duration, content, recency, intensity, pedagogical approach, or clinical integration of the training received. Recent evidence suggests that the characteristics and structure of spiritual care education may be important for developing competence. For example, structured and experiential educational interventions, including scenario-based learning and competency-based programmes, have been shown to improve nurses’ self-assessed spiritual care competence [51,52]. Similarly, a recent quasi-experimental study demonstrated significant improvements in spiritual care competencies following a competency-based educational programme incorporating the EPICC Spiritual Care Education Standard [53]. These findings highlight the importance of examining the structure, content, intensity, experiential components, and clinical integration of education rather than training status alone. Accordingly, the absence of a significant association between spiritual care training and SCCS scores in the present study should not be interpreted as evidence that spiritual care education is ineffective. Rather, it may indicate that a simple yes/no training variable was insufficient to capture the educational exposure necessary to influence spiritual care competence. Therefore, the educational implications of the present findings should focus not simply on increasing access to spiritual care training, but on improving the quality, structure, experiential components, and clinical integration of such education. Future studies should examine spiritual care education in greater detail, including its duration, timing, theoretical and experiential content, teaching strategies, opportunities for supervised practice, and integration into clinical settings. A similar caution applies to palliative care education, for which the absence of a significant difference in the present study should not be interpreted as evidence that such education is ineffective, but rather as an indication that a simple training-status variable was not associated with the measured outcomes in this sample.

From a nursing complexity perspective, these findings suggest that individual competency development and the organization of care should be considered as interconnected aspects of palliative nursing practice, although the present study did not directly measure or test their relative contributions. Educational programmes may strengthen spiritual care knowledge and communication skills through case-based learning, reflection, supervised practice, and integration of spiritual assessment into routine care. At the same time, future studies should examine how staffing, teamwork, time, patient acuity, and institutional support interact with individual competencies in shaping nursing practice. Recent research on nursing complexity emphasizes that care activities are interconnected and that the complexity of nursing work is shaped by both the characteristics of care activities and the context in which they are performed [46,47,48]. Intervention and implementation studies could therefore investigate whether competency development combined with supportive practice environments is associated with objectively assessed care processes or patient and family reported outcomes.

Finally, the regression model explained only a limited proportion of the variance in perceived quality of palliative nursing care. This finding suggests that the outcome is likely influenced by multiple factors that were not assessed in the present study. However, because organizational conditions, workload, institutional support, and other contextual variables were not directly measured or included in the model, their relative contribution cannot be determined from the current findings. In addition, institution-level clustering could not be assessed because hospital identifiers were unavailable in the dataset. Therefore, potential similarities among nurses working within the same institution could not be accounted for in the analyses. No missing data were present in the final analytic dataset because incomplete responses were excluded before analysis. Future studies should complement self-reported measures with the analysis of nursing documentation, since spiritual and relational interventions may remain hidden within heterogeneous free-text records; natural language processing and semantic mapping may help identify and standardize these otherwise under-recognized nursing activities [44]. Future research should therefore use longitudinal, mixed-method, and multimethod designs with more diverse samples and incorporate individual-, clinical-, organizational-, and patient-level factors to provide a more comprehensive understanding of perceived quality of palliative nursing care.

### 4.1. Strengths

This study has several strengths. First, it addresses a relatively underexplored area by examining the association between nurses’ spiritual care competence and their perceived quality of palliative nursing care within the Turkish cultural context. Second, the use of valid and reliable measurement instruments strengthens the methodological rigor of the study. Third, the inclusion of nurses working in different clinical settings, including palliative care, oncology, and intensive care units, contributes to the clinical diversity of the sample and provides perspectives from different contexts in which palliative care may be delivered. Finally, the study provides relevant insights for nursing practice and education by considering spiritual care competence as one component of holistic palliative nursing practice and by highlighting the conceptual relationship between spiritual care competence and perceived quality of palliative nursing care.

### 4.2. Limitations

This study has several limitations that should be considered when interpreting the findings. First, the cross-sectional design does not allow causal inferences or the assessment of changes over time; therefore, the observed association between spiritual care competence and perceived quality of palliative nursing care should be interpreted as correlational rather than causal. Second, the use of convenience sampling and recruitment through a professional nursing association may have introduced selection bias and may limit the generalizability of the findings. Nurses who participate in professional associations or voluntarily respond to online surveys may differ from non-participants in terms of professional engagement, motivation, or interest in palliative and spiritual care.

Third, both study variables were assessed using self-report instruments, which may be subject to response and social desirability biases. In addition, self-reported spiritual care competence may not fully reflect how consistently spiritual care is incorporated into everyday nursing practice. Future studies could therefore complement self-reported measures with objective or practice-based indicators, such as nursing documentation, observational measures, or patient-reported assessments. Fourth, online data collection may have limited participation among nurses with restricted access to or familiarity with digital technologies. Although the use of validated instruments strengthens measurement reliability, the exclusive use of quantitative self-report data limited the assessment of the contextual and experiential factors underlying the observed findings.

Finally, the regression model explained only a limited proportion of the variance in perceived quality of palliative nursing care. This finding suggests that the outcome is likely influenced by multiple factors that were not assessed in the present study. However, because organizational conditions, workload, institutional support, and other contextual variables were not directly measured or included in the model, their relative contribution cannot be determined from the current findings. In addition, institution-level clustering could not be assessed because hospital identifiers were unavailable in the dataset. Therefore, potential similarities among nurses working within the same institution could not be accounted for in the analyses. No missing data were present in the final dataset, as responses with incomplete data were excluded before analysis. Future research should therefore use longitudinal, mixed-method, and multimethod designs with more diverse samples and incorporate individual-, clinical-, organizational-, and patient-level factors to provide a more comprehensive understanding of perceived quality of palliative nursing care.

## 5. Conclusions

This study found that nurses reported relatively high levels of perceived quality of palliative nursing care and spiritual care competence. A weak positive statistical association was observed between spiritual care competence and perceived quality of palliative nursing care. Because both constructs were assessed using self-reported measures in a cross-sectional design, this association should not be interpreted as evidence that spiritual care competence improves or determines the quality of palliative nursing care.

Postgraduate education was associated with higher perceived quality of palliative nursing care. These cross-sectional differences should not be interpreted as evidence that postgraduate education improves care quality, as the observed association may reflect other individual or professional characteristics, such as clinical experience, professional role, or clinical setting, which were not fully accounted for in the present study.

Overall, the findings suggest that perceived quality of palliative nursing care and spiritual care competence are related but conceptually distinct aspects of nursing practice. The modest explanatory power of the regression model further indicates that substantial variability in perceived quality of palliative nursing care remained unexplained by the variables included in the analysis. Future longitudinal and multimethod studies should incorporate individual, clinical, organizational, and patient-level factors and use objective and patient-centered indicators to better identify the determinants of perceived palliative nursing care quality.

## Figures and Tables

**Table 1:** Socio-demographic and clinical data of the participants (N = 160).

Characteristics	M ± SD (Median)	Min.–Max.	
Age (years)	30.43 ± 8.95 (28.0)	20–50	
Palliative nursing experience (years)	5.96 ± 3.83 (6.0)	1–12	
Parameters		N	%
Gender	Female	124	77.5
	Male	36	25.5
Marital status	Married	88	55.0
	Single	72	45.0
Educational Level	Associate degree or less	32	20.0
	Bachelor’s degree	104	65.0
	Postgraduate degree	24	15,0
Palliative nursing experience (years)	1–5	45	28.0
	6–10	57	35.6
	11+	58	36.3
Palliative care training	Yes	79	49.4
	No	81	50.6
Spiritual care training	Yes	35	21.9
	No	125	78.1
Spiritual care competence	Sufficient	35	21.9
	Not enough	125	78.1
Palliative care unit type	Palliative unit	69	43.1
	Oncology	41	25.6
	Adult intensive care	31	19.4
	Pediatric intensive care	19	11.9
Barriers to spiritual care	Information lack	22	13.8
	Inapplicability	18	11.3
	Lack of time	26	16.3
	Nurse shortage	26	16.3
	High patient load	38	23.8
	Task-oriented	30	18.8

M: mean; SD: standard deviation; Min: minimum; Max: maximum; N: count/frequency; %: percent.

**Table 2:** Comparison of personal and working conditions according to scales (PNCQS & SCCS).

		PNCQS			SCCS		
		M ± SD	Test	*p* (d/η^2^)	M ± SD	Test	*p*
Age (r)			−0.06	0.44		−0.122	0.13
Gender (t)							
	Female	76.3 ± 9.3	2.778 ^a^	0.006 *	93.3 ± 12.6	1.031	0.30
	Male	71 ± 12		(0.53)	90.9 ± 10.2		
Marital status (t)							
	Married	74.7 ± 9.8	−0.455	0.65	91.9 ± 12.2	−0.957	0.34
	Single	75.5 ± 10.6			93.8 ± 12		
Educational level (F)							
	Associate degree or less ^A^	70.8 ± 8.9	25.0 ^b^	<0.001	95.6 ± 12.4	1.22	0.3
	Bachelor’s degree ^A^	74.9 ± 10.8	^B^ > ^A^ **	(0.10)	91.8 ± 12.2		
	Postgraduate degree ^B^	81.6 ± 3.8			93.2 ± 11.1		
Palliative nursing experience (Years) (F)							
	1–5	75.7 ± 9.6	1.122	0.328	91.2 ± 11.5	2.33	0.101
	6–10	73.5 ± 10.1			95.0 ± 10.1		
	11+	76.2 ± 10.6			95.8 ± 11.5		
Palliative care training (t)							
	Yes	76.4 ± 11.5	1.68 ^a^	0.095	92.2 ± 12.1	−0.654	0.51
	No	73.7 ± 8.4			93.4 ± 12.1		
Spiritual care training (t)							
	Yes	73.5 ± 12.4	−1.071 ^a^	0.362	91.9 ± 11.7	−0.456	0.657
	No	75.4 ± 9.4			92.9 ± 12.3		
Spiritual care competence (t)							
	Sufficient	76.6 ± 7.6	1.233 ^a^	0.221	94.7 ± 10.4	1.101	0.272
	Not enough	74.6 ± 10.7			92.2 ± 12.5		
Palliative care unit type (F)							
	Palliative	74.3 ± 10.1	1.681	0.173	93.6 ± 11.2	0.75	0.52
	Oncology	73.8 ± 10.8			93.5 ± 13.6		
	Adult ICU	75.6 ± 10.6			89.9 ± 12.7		
	Pediatric ICU	79.7 ± 6.8			92.7 ± 11.4		
Barriers to spiritual care (F)							
	Information lack	74 ± 11.2	1.337	0.251	91.4 ± 11.9	1.074	0.377
	Inapplicability	76.6 ± 10.9			98.8 ± 11.2		
	Lack of time	73.2 ± 9.5			91.7 ± 11.5		
	Nurse shortage	76.3 ± 11.5			91.3 ± 13.4		
	High patient load	77.7 ± 9.7			92.8 ± 11.5		
	Task-oriented	72.3 ± 8.3			92.2 ± 12.8		

M: mean; SD: standard deviation; r: Pearson correlation; t: independent samples *t*-test (^a^ Levene t < 0.05, d: Cohen’s d); F: one-way ANOVA (^b^ Levene test < 0.05, η^2^: Eta square); ** post hoc (Games–Howell). Means with the same letter are not statistically significantly different (*p* > 0.05), * *p* < 0.05.

**Table 3:** The relationship between the total scores and subscales of the Palliative Care Quality Scale and the Spiritual Care Competence Scale.

	M ± SD		PNCQS	SCCS	EAIOSC	PAPCISC	ATPSAC
PNCQS	75.08 ± 10.15	r:	1	0.161 *	0.121	0.155	0.067
		*p*:		0.042	0.129	0.051	0.399
SCCS	92.8 ± 12.1	r:		1	0.443 **	0.774 **	0.542 **
		*p*:			0.000	0.000	0.000
EAIOSC	20.9 ± 3.3	r:			1	0.271 **	0.205 **
		*p*:				0.003	0.027
PAPCISC	50.8 ± 11.2	r:				1	0.387 **
		*p*:					0.000
ATPSAC	22.5 ± 4.7	r:					1

M: mean; SD: standard deviation; r: Pearson correlation coefficient, * *p* < 0.05, ** *p* < 0.001; EAIOSC: evaluation and implementation of spiritual care sub-dimension; PAPCISC: professionalism and patient counseling sub-dimension in spiritual care; ATPSAC: attitude towards the patient’s spirituality and communication sub-dimension.

**Table 4:** Multiple linear regression analysis for PNCQS.

	B	SE	LB	UB	β	t	*p*
(Constant)	63.194	5.97	51.5	74.9		10.595	<0.001
Spiritual Care Competency Scale	0.127	0.063	0.004	0.251	0.153	2.027	0.044 *
Gender (Ref::Female, 1:Male)	−5.182	1.81	−8.73	−1.63	−0.214	−2.862	0.005 *
Educational level (Ref::High school, Associate degree, Bachelor’s degree; 1: Postgraduate degree)	8.813	2.13	4.64	13.0	0.311	4.145	<0.001
Model Summary R: 0.388, R^2^: 0.150, R^a^: 0.134, SE: 9.4, F: 9.192, df1: 3, df2: 156, *p* < 0.001, DW: 2.068, VIF: 1.025–1.052, Tolerance: 0.951–0.976 Std.Residual Min–Max [−2.47, 1.85], Mean: [0.000], sd: 0.99							

B: unstandardized coefficients; SE: standard error; LB–UB: lower–upper bound, 95.0% confidence interval for B; β: standardized coefficients; t: independent samples *t*-test, * < 0.05; RC: reference category; Ref: reference category; R: multiple correlation coefficient; R^2^: coefficient of determination; R^a^: adjusted R^2^; SE: standard error of the estimate; F: ANOVA, df: degrees of freedom.

## Data Availability

The data presented in this study are not publicly available due to privacy and confidentiality considerations related to participant data. An anonymized version of the dataset is available from the corresponding author upon reasonable request.

## Supplementary Materials

The following supporting information can be downloaded at: https://www.mdpi.com/article/10.3390/healthcare14172796/s1, Supplementary File: Supplementary Material S1 and Supplementary Material S2. Supplementary Material S1: In order to avoid over-parameterization and to establish the most parsimonious and explanatory model, the Principle of Parsimony was adopted in the study. Accordingly, the Full Model (Saturated Model), which included all collected independent variables (spiritual care training, self-perceived competence, and clinical unit), was entered into the analysis. All estimated coefficients and regression diagnostics obtained from this model are presented below (Table S1). In accordance with the two-stage empirical variable selection approach (data-driven/empirical confounder selection) and the principle of parsimony adopted in the study, the main regression model was constructed using only those predictors that demonstrated a statistically significant association/difference with the dependent variable in the preliminary analyses. Indeed, examination of the full model (Saturated Model) revealed that SCCS (*p* = 0.037), sex (*p* = 0.002), and educational level (*p* = 0.001) were statistically significant predictors of PCQS. These findings were fully consistent with the results identified in the preliminary analyses for SCCS (Table 3 in the main text), sex, and educational level (Table 2 in the main text). Conversely, variables that were non-significant in the preliminary analyses and did not make a statistically meaningful contribution to the full model (such as spiritual care training, self-perceived competence, and clinical unit) were excluded. This approach both prevented over-parameterization and yielded the most parsimonious main model with the highest explanatory power. Assessment of Regression Assumptions, In addition, to assess the accuracy and reliability of the regression analysis, advanced multiple regression assumptions were examined beyond univariate normality tests (kurtosis/skewness): Independence of Observations (Autocorrelation): The Durbin–Watson statistic was 2.170, indicating that there was no evidence of an autocorrelation problem. Multicollinearity: The VIF (Variance Inflation Factor) values for all independent variables ranged from 1.096 to 2.214 (all values < 5.0), confirming that there was no substantial risk of multicollinearity. Outliers and Influential Observations: Standardized residuals ranged from −2.945 to 1.921, remaining within the critical ±3.0 range. Thus, no extreme observation capable of distorting the model was identified. Normality of Error Terms and Linearity: The histogram of the residuals (Figure S1) and the Normal P-P plot (Figure S2) indicated that the error terms exhibited an approximately normal distribution and that the assumption of linearity was adequately satisfied. Supplementary Material S2: To maintain the readability and concise nature of the main manuscript, we have summarized the key residual diagnostic metrics (Std. Residual Min-Max [−2.47, 1.85], Mean: [0.000], SD: 0.99) directly in the footnote of the main regression table and explained them within the text. However, to ensure full visual and methodological transparency, the corresponding diagnostic plots (Histogram and Normal P-P plot) for the final model have been provided in the Supplementary Material (Figures S3 and S4).
