# Supplementary material for: Association Between Spiritual Care Competence and Palliative Care Quality Among Palliative Care Nurses: A Cross-Sectional Study
Source: Healthcare (Basel). 2026 Sep 1;14(17):2796. doi: 10.3390/healthcare14172796 (PMC13565115; doi:10.3390/healthcare14172796)
Supplement: Supplementary file 1 [file healthcare-14-02796-s001.zip › healthcare-4482342-supplementary.pdf]

Supplementary Material S1

In order to avoid over-parameterization and to establish the most parsimonious and explanatory model, the **Principle of Parsimony** was adopted in the study. Accordingly, the **Full Model (Saturated Model)**, which included all collected independent variables (spiritual care training, self-perceived competence, and clinical unit), was entered into the analysis. All estimated coefficients and regression diagnostics obtained from this model are presented below (Table S1).

Table S1. Full Saturated Multiple Linear Regression Model and Diagnostic Statistics (N=160)

	**Unstandardized Coefficients**		**Standardized Coefficients**	**t**	**Sig.**	**Collinearity Statistics**	
	**B**	**Std. Error**	**Beta**			**Tolerance**	**VIF**
(Constant)	63.492	6.769		9.38	0,000		
**Spiritual Care Competency Scale**	**0.135**	**0.064**	**0.161**	**2.1**	**0.037**	0.912	1.096
**Gender (1=Male)**	**-6.293**	**2.009**	**-0.26**	**-3.133**	**0.002**	0.78	1.282
Marital (1=Single)	-0.311	1.892	-0.015	-0.164	0.87	0.619	1.615
**Educational (1=PDG)**	**7.7**	**2.271**	**0.272**	**3.39**	**0.001**	0.834	1.199
Experience (1=1-5)	-1.661	2.452	-0.074	-0.677	0.499	0.452	2.214
Experience (1=6-10)	-2.945	1.977	-0.139	-1.49	0.139	0.612	1.633
PCT (1=Yes)	0.723	1.964	0.036	0.368	0.713	0.569	1.757
SCT (1=Yes)	-0.831	2.408	-0.034	-0.345	0.731	0.554	1.805
SC Competance (1=Sufficient)	1.272	2.204	0.052	0.577	0.565	0.661	1.513
PC Unit (1=Oncology)	-0.345	2.094	-0.014	-0.165	0.869	0.765	1.308
PC Unit (1=Adult intensive care)	3.919	2.149	0.153	1.823	0.070	0.761	1.315
PC Unut (1=Pediatric intensive care)	3.387	2.217	0.125	1.528	0.129	0.796	1.256
Barrier (1=Information Lack)	-1.217	2.745	-0.041	-0.443	0.658	0.614	1.628
Barrier (1=Inapplicability)	0.43	2.965	0.013	0.145	0.885	0.625	1.6
Barrier (1=Lack of time)	-2.206	2.604	-0.08	-0.847	0.398	0.595	1.681
Barrier (1=Nurse shortage)	3.009	2.649	0.11	1.136	0.258	0.575	1.74
Barrier (1=Task-Oriented)	-3.858	2.513	-0.149	-1.535	0.127	0.57	1.753

Note 1: Dependent Variable: Palliative Care Quality Scale. Model Fit: (R=.489), (R2=.239), (Adjusted R2=.148), (F(17, 142)=2.622, p=.001).
Note 2 (Multivariate Diagnostics): Durbin-Watson=2.170 (No autocorrelation). Standardized Residuals=Minimum: -2.945, Maximum: 1.921 (No influential outliers exceeding ±3.0). Multicollinearity is absent as all VIF values <2.22).
Note 3 (Reference Categories for Dummy Variables): 'Experience (1=+11)', 'PC Unit (1=Palliative unit)', and 'Barrier (1=High patient load)' were utilized as reference groups and automatically excluded by the system to avoid the dummy variable trap

In accordance with the two-stage empirical variable selection approach (**data-driven/empirical confounder selection**) and the principle of parsimony adopted in the study, the main regression model was constructed using only those predictors that demonstrated a statistically significant association/difference with the dependent variable in the preliminary analyses. Indeed, examination of the full model (**Saturated Model**) revealed that **SCCS (p = .037), sex (p = .002), and educational level (p = .001)** were statistically significant predictors of PCQS. These findings were fully consistent with the results identified in the preliminary analyses for SCCS (Table 3 in the main text), sex, and educational level (Table 2 in the main text). Conversely, variables that were non-significant in the

preliminary analyses and did not make a statistically meaningful contribution to the full model (such as spiritual care training, self-perceived competence, and clinical unit) were excluded. This approach both prevented **over-parameterization** and yielded the most parsimonious main model with the highest explanatory power.

Assessment of Regression Assumptions

In addition, to assess the accuracy and reliability of the regression analysis, advanced multiple regression assumptions were examined beyond univariate normality tests (kurtosis/skewness):

Independence of Observations (Autocorrelation): The Durbin–Watson statistic was **2.170**, indicating that there was no evidence of an autocorrelation problem.

Multicollinearity: The VIF (Variance Inflation Factor) values for all independent variables ranged from **1.096 to 2.214** (all values < 5.0), confirming that there was no substantial risk of multicollinearity.

Outliers and Influential Observations: Standardized residuals ranged from **−2.945 to 1.921**, remaining within the critical ±3.0 range. Thus, no extreme observation capable of distorting the model was identified.

Normality of Error Terms and Linearity: The histogram of the residuals (**Figure S1**) and the Normal P–P plot (**Figure S2**) indicated that the error terms exhibited an approximately normal distribution and that the assumption of linearity was adequately satisfied.

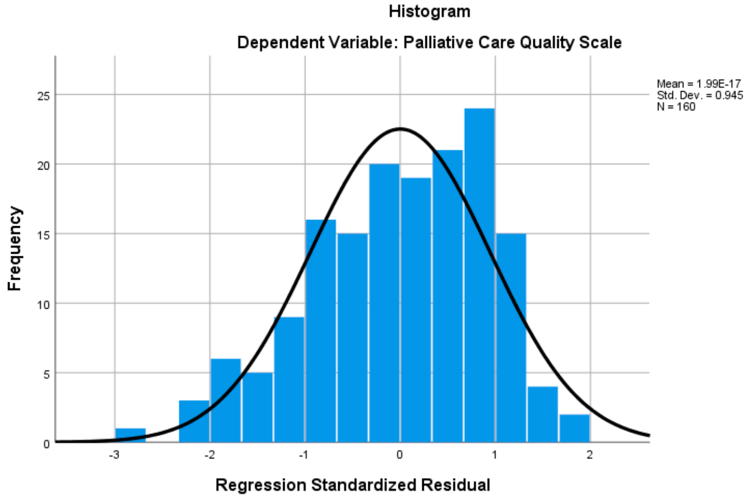

Figure S1

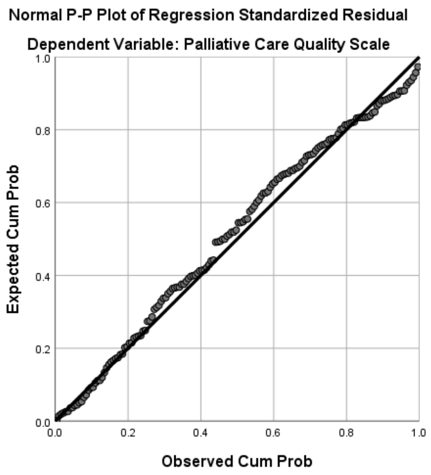
Normal P-P Plot of Regression Standardized Residual
Dependent Variable: Palliative Care Quality Scale
Expected Cum Prob
Observed Cum Prob
0.0
0.2
0.4
0.6
0.8
1.0

Figure S2

Supplementary Material S2

To maintain the readability and concise nature of the main manuscript, we have summarized the key residual diagnostic metrics (Std. Residual Min-Max [-2.47, 1.85], Mean: [.000], SD: .99) directly in the footnote of the main regression table and explained them within the text. However, to ensure full visual and methodological transparency, the corresponding diagnostic plots (Histogram and Normal P-P plot) for the final model have been provided in the **Supplementary Material (Figure S3 and Figure S4)**

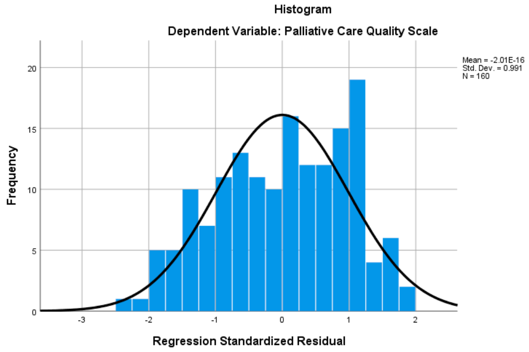

Figure S3

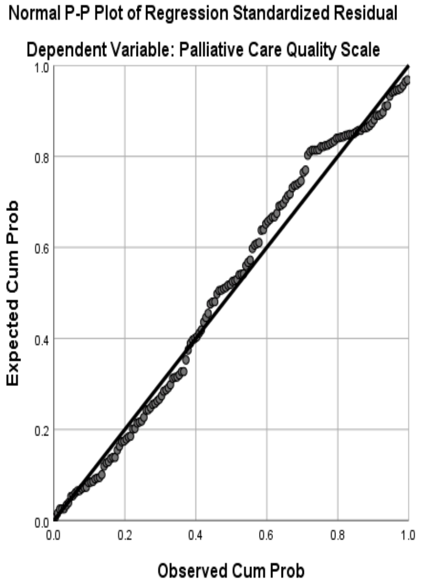

Figure S4
